# Calcium Signaling and Mitochondrial Function in Presenilin 2 Knock-Out Mice: Looking for Any Loss-of-Function Phenotype Related to Alzheimer’s Disease

**DOI:** 10.3390/cells10020204

**Published:** 2021-01-21

**Authors:** Alice Rossi, Luisa Galla, Chiara Gomiero, Lorena Zentilin, Mauro Giacca, Valentina Giorgio, Tito Calì, Tullio Pozzan, Elisa Greotti, Paola Pizzo

**Affiliations:** 1 Department of Biomedical Sciences, University of Padua, 35131 Padua, Italy; Alice.Rossi@mdc-berlin.de (A.R.); luisa.galla@unipd.it (L.G.); chiara.gomiero@unipd.it (C.G.); vgiorgio@bio.unipd.it (V.G.); tito.cali@unipd.it (T.C.); tullio.pozzan@unipd.it (T.P.); paola.pizzo@unipd.it (P.P.); 2 Neuroscience Institute, National Research Council (CNR), 35131 Padua, Italy; 3 International Centre for Genetic Engineering and Biotechnology (ICGEB), 34149 Trieste, Italy; lorena@icgeb.org (L.Z.); giacca@icgeb.org (M.G.); 4 Department of Biomedical and Neuromotor Science, University of Bologna, 40112 Bologna, Italy; 5 Venetian Institute of Molecular Medicine (VIMM), 35131 Padua, Italy

**Keywords:** Alzheimer′s disease, presenilin 2, PS2–/–, Ca^2+^ signaling, mitochondria, bioenergetics, mitochondrial membrane potential, oxygen consumption rate, neuronal hyperexcitability

## Abstract

Alzheimer′s disease (AD) is the most common age-related neurodegenerative disorder in which learning, memory and cognitive functions decline progressively. Familial forms of AD (FAD) are caused by mutations in amyloid precursor protein (*APP*), presenilin 1 (*PSEN1*) and presenilin 2 (*PSEN2*) genes. Presenilin 1 (PS1) and its homologue, presenilin 2 (PS2), represent, alternatively, the catalytic core of the γ-secretase complex that, by cleaving APP, produces neurotoxic amyloid beta (Aβ) peptides responsible for one of the histopathological hallmarks in AD brains, the amyloid plaques. Recently, *PSEN1* FAD mutations have been associated with a loss-of-function phenotype. To investigate whether this finding can also be extended to *PSEN2* FAD mutations, we studied two processes known to be modulated by PS2 and altered by FAD mutations: Ca^2+^ signaling and mitochondrial function. By exploiting neurons derived from a *PSEN2* knock-out (PS2–/–) mouse model, we found that, upon IP_3_-generating stimulation, cytosolic Ca^2+^ handling is not altered, compared to wild-type cells, while mitochondrial Ca^2+^ uptake is strongly compromised. Accordingly, PS2–/– neurons show a marked reduction in endoplasmic reticulum–mitochondria apposition and a slight alteration in mitochondrial respiration, whereas mitochondrial membrane potential, and organelle morphology and number appear unchanged. Thus, although some alterations in mitochondrial function appear to be shared between PS2–/– and FAD-PS2-expressing neurons, the mechanisms leading to these defects are quite distinct between the two models. Taken together, our data appear to be difficult to reconcile with the proposal that FAD-PS2 mutants are loss-of-function, whereas the concept that PS2 plays a key role in sustaining mitochondrial function is here confirmed.

## 1. Introduction

About 8% of the world’s population with ages ≥65 years are affected by dementia, considerably impacting on global healthcare and social/economic costs. Alzheimer’s Disease (AD) contributes to the majority of dementia cases and is characterized by a progressive accumulation of cognitive dysfunctions, partially due to the degeneration of the cholinergic and glutamatergic systems, which include both altered levels of neurotransmitters and disruption of neuronal networks [1]. Most AD cases are sporadic (SAD), while only a small percentage are genetic and run in families (Familial AD—FAD). The overlapping phenotype between the two forms allows researchers to exploit FAD mouse models to study AD pathogenesis and test possible treatments for contrasting the disease.

The FAD-linked mutations are located in the amyloid precursor protein (*APP*), presenilin 1 (*PSEN1*) and presenilin 2 (*PSEN2*) genes, with *PSEN1* and *PSEN2* mutations accounting for more than 80% of genetic FAD [2]. Presenilin 1 (PS1) and its homologue presenilin 2 (PS2), constitute, alternatively, the catalytic core of the γ-secretase complex that, by cleaving APP in concert with β-secretase, produces neurotoxic amyloid beta (Aβ) peptides. Aβ peptide aggregates form brain extracellular amyloid plaques that, together with intracellular neurofibrillary tangles of hyperphosphorylated tau, represent the histopathological hallmarks of AD [3].

The mechanism linking FAD mutations to neuronal dysfunction and, ultimately, cell death is still largely obscure. Importantly, no treatments are available for this pathology and many clinical trials of drugs targeting Aβ have failed, even though some new drugs are under investigation [4].

In this complex scenario, the recently proposed hypothesis of a loss-of-function phenotype associated with FAD-linked PS1 mutations raises new questions about AD pathogenesis. In particular, it has been suggested that FAD-PS1 mutations drastically reduce γ-secretase activity, causing: (i) an overall decrease in the production of Aβ peptides, despite increasing the Aβ42/Aβ40 ratio, and thus exacerbating Aβ deposition; (ii) memory impairment and synaptic dysfunction [5].

Considering the similar phenotype characterizing AD patients carrying PS1 or PS2 mutations, as well as SAD patients, and the similar increased Aβ deposition in mouse models expressing either FAD-PS1 or FAD-PS2 (usually in combination with human FAD-APP), it is tempting to speculate that if the loss-of-function pathogenic hypothesis is correct for FAD-PS1 models, it should be also valid for FAD-PS2-linked forms.

In order to address this point at the cellular and molecular levels, we took advantage of the PS2–/– mouse model [6], knock-out (KO) for *PSEN2*. Using PS2–/– primary neuronal cultures, we investigated specific features (Ca^2+^ homeostasis and mitochondrial function) previously characterized in FAD-PS2-expressing cells.

As far as cytosolic Ca^2+^ handling is concerned, we found that PS2–/– neurons, both cortical and hippocampal, in response to a metabotropic stimulation, are indistinguishable from wild-type (WT) cells, indicating no alteration in endoplasmic reticulum (ER) Ca^2+^ content, in contrast to what has been observed in cells expressing FAD-PS2 mutants, showing, consistently, a reduction in this parameter [7,8,9,10,11,12,13,14,15]. On the contrary, mitochondrial [Ca^2+^] transients, under the same conditions, are significantly reduced in PS2–/– cells, mainly due to a substantial reduction in the physical apposition between the ER and mitochondria. Moreover, we observed that PS2–/– neurons display a slight reduction in mitochondrial respiration, only partially resembling some mitochondrial features found in FAD-PS2-expressing neurons [16,17]. Similarly, the absence of PS2 is associated with neuronal hyperexcitability, as already showed for a FAD-PS2 mutant [12]. Altogether, these observations demonstrate that, although PS2–/– neurons present some features shared with FAD-PS2-expressing neurons, the mechanisms leading to these phenotypes, at least for the mitochondrial one, are clearly distinct. Our data indicate that the mitochondria-related phenotypes associated with FAD-PS2 mutations are not linked to a protein loss-of-function and suggest that PS2 plays key and multiple roles in controlling mitochondrial function.

## 2. Materials and Methods

### 2.1. Animals

All procedures were conducted in accordance with the Italian and European Communities Council Directive on Animal Care and were approved by the Italian Ministry of Health. Handling of animals was in accordance with Directive 2010/63/EU of the European Parliament on the protection of animals used for scientific purposes, with the explicit approval of the local veterinary authority (Project code: D2784.N.HEH).

### 2.2. Primary Neuronal Cultures

Primary neuronal cultures were obtained from cortices or hippocampi, dissected from 0- to 1-day-old newborn mice as previously described [12]. The transgenic mouse line PS2–/– was used (Infrafrontier, Monterotondo Scalo, Rome, Italy). The line has the background strain of C57BL/6 mice, which were used as WT controls (Charles River, Wilmington, MA, USA).

Mouse line features:

- (1) The PS2–/– are homozygous deficient mice, where the *PSEN2* gene was inactivated, replacing the mouse PS2 exon 5 with hygromycin cassette under the control of the PGK promoter [6];
- (2) The C57BL/6J WT mice share >90% genetic background of the other line.

Cells were seeded on coated coverslips (poly-L-lysine, 100 µg/mL for cortical; poly-L-lysine 30 μg/mL and laminin 2 μg/mL for hippocampal neurons) with the following densities:

- 3 × 10^5^ cells/well, for colocalization experiments;
- 8 × 10^5^ cells/well for FRET experiments;
- 3 × 10^5^ cells/well for Seahorse experiments;
- 8 × 10^5^ cells/well for tetramethyl rhodamine methyl ester (TMRM) experiments;
- 1.5 × 10^6^ cells/well for Western Blot and Nonyl Acridine Orange (NAO) experiments.

This was performed in MEM (Gibco, 32360026, ThermoFisher Scientific, Waltham, MA, USA) containing glucose (20 mM), L-glutamine (0.5 mM), B27 supplement (0.5%), N2 supplement (1%), pyruvic acid (1 mM), biotin (3.6 µM), penicillin (25 μg/mL), streptomycin (25 μg/mL), neomycin (50 μg/mL) and horse serum (10%). Twenty-four hours after plating, the medium was replaced with serum- and antibiotic-free with BME (Gibco, 41010026) supplemented with B27 (2%), L-glutamine (0.5 mM) and sodium pyruvate (0.23 mM). All the experiments were performed at 14–16 days in vitro (DIVs).

### 2.3. Adeno-Associated Virus (AAV) Production

Recombinant AAV vectors used in this study were prepared by the AAV Vector Unit at the International Center for Genetic Engineering and Biotechnology Trieste (http://www.icgeb.org/avucore-facility.html), as described previously [18] with a few modifications. Briefly, infectious recombinant AAV vector particles were generated in HEK293T cultures in roller bottles by a cross-packaging approach whereby the vector genome was packaged into AAV capsid serotype-9 (AAV9) [19]. Viral stocks were obtained by PEG precipitation and CsCl_2_ gradient centrifugation [20]. The physical titer of recombinant AAVs was determined by quantifying vector genomes (vgs) packaged into viral particles, by real-time PCR against a standard curve of a plasmid containing the vector genome [21]; values obtained were in the range of 1 × 10^12^ to 1 × 10^13^ vg per milliliter.

### 2.4. Ca^2+^ Imaging

Neurons were infected with AAV9-human-synapsin (hSyn)-4mtµ+16 or AAV9-hSyn-D3mCerulean3+16 and the experiments were performed 7 days after transduction. Cortical or hippocampal neurons (DIVs 14–16) expressing FRET probes were analyzed using a DM6000 inverted microscope (Leica, Wetzlar, Germany) and a 40 x oil objective (HCX Plan Apo, NA 1.25). Excitation light, produced by a 405 nm LED (Led Engin #LZ1-00 UA00 LED), was filtered at the appropriate wavelength (425 nm) through a band pass filter, and the emitted light was collected through a beam splitter (OES s.r.l., Padua, Italy) (emission filters HQ 480/40M (for CFP) and HQ 535/30M (for YFP); dichroic mirror 515 DCXR). All filters and dichroics were from Chroma Technologies (Bellow Falls, VT, USA). Images were collected using an IM 1.4C cool camera (Jenoptik Optical Systems, Jena, Germany) attached to a 12-bit frame grabber. Synchronization of the excitation source and the camera was performed through a control unit run by a custom-made software package, Roboscope (developed by Catalin Dacian Ciubotaru at VIMM, Padua, Italy). During the experiments, cells were mounted into an open-topped chamber and kept at 37 °C. Images were acquired every 1 s, with 300 ms exposure, for KCl and metabotropic stimulation experiments. Images were acquired every 500 ms, with 300 ms exposure, for picrotoxin stimulation experiments.

Neurons were infected with AAV9-hSyn-4mtGCaMP6f and the experiments were performed 7 days after virus addition. Cortical neurons (DIVs 14–16) expressing 4mtGCaMP6f probes were analyzed using 40× objectives (Olympus Biosystems GmbH, Planegg, Germany) on an Axiovert 100 inverted microscope (Zeiss, Jena, Germany). Alternated excitation wavelengths of 470 and 410 nm were obtained by a monochromator (Polychrome V, TILL-Photonics) controlled by Roboscope. The emitted fluorescence was measured at 500–530 nm. Images were acquired every 5 s, with a 300 ms exposure time at 470 nm and 500 ms at 410 nm, by a PCO SensiCam QE camera (Kelheim, Germany) controlled by the same software. Regions of interest (ROIs), corresponding to the entire soma, were selected for Ca^2+^ imaging.

Cells were perfused with mKRB (in mM: 140 NaCl, 2.8 KCl, 2 MgCl_2_, 10 Hepes, 1 CaCl_2_, 5 glucose; pH 7.4 at 37 °C) supplemented as detailed below.

- In Ca^2+^ experiments, upon metabotropic stimulation, neurons were perfused with: (1) mKRB, 60 s; (2) mKRB supplemented with EGTA (600 μM) for 20 s; (3) mKRB supplemented with CPA (20 μM), carbachol (500 μM); glutamate (100 μM) and ATP (100 μM).
- In Ca^2+^ experiments upon KCl stimulation, neurons were perfused with: (1) mKRB, 60 s; (2) mKRB supplemented with KCl (30 mM) for 170 s; (3) mKRB supplemented with EGTA (600 μM) for 200 s. KCl stimulation was performed in a modified mKRB to maintain the osmolarity. In particular, the KCl-based mKRB solution contains, in mM: 110 NaCl, 30 KCl, 2 MgCl_2_, 10 Hepes, 1 CaCl_2_, 5 glucose; pH 7.4 at 37 °C.
- In picrotoxin Ca^2+^ experiments, upon picrotoxin stimulation, neurons were continuously perfused with mKRB supplemented with picrotoxin (25 μM) for 100 s.
- In Ca^2+^ experiments, to evaluate mitochondrial resting Ca^2+^ level with 4mtGCaMP6f, 3–5 fields of view per coverslip were imaged for 1 min, keeping cells at 37 °C and in mKRB supplemented with 1 mM CaCl_2_.

Pseudocolor images have been generated using ImageJ. Briefly, the plugin image calculator has been used to create ratio images and then the lookup table function “spectrum” has been used to create the pseudocolor images.

### 2.5. Oxygen Consumption Rate (OCR) and Extracellular Acidification Rate (ECAR) Measurements

Cortical neurons were seeded in 500 μL of BME medium (see above) in XF24 cell culture microplates (Seahorse Bioscience, Agilent, Santa Clara, CA, USA). At DIVs 14–16, the medium was replaced with 670 μL of *seahorse medium* (DMEM D5030 supplemented with, in mM: 1 sodium pyruvate; 5 glucose; 0.031 NaCl, 0.5 glutamine; pH 7.4 at 37 °C). Cells were incubated at 37 °C for 1 h, and then OCR and ECAR were measured with an XF24 Extracellular Flux Analyzer (Seahorse Bioscience). After OCR baseline measurement, specific drugs were added as indicated (oligomycin-A, 1 μg/mL); carbonyl cyanide-4-(trifluoromethoxy)phenylhydrazone (FCCP, 0.5 μM, defined after titration); rotenone (1 μM) and antimycin A (0.1 μM). Both the OCR and extracellular acidification rate (ECAR) were considered.

The classical “mitochondrial stress test” protocol was performed as previously described [22]. After calculation of the buffering power of the experimental saline and the proton rate production [23], mitochondrial and cytosolic (glycolytic) ATP production rates were calculated from OCR and ECAR measurements as described previously [24].

### 2.6. Preparation of Protein Extracts and Western Blot Analysis

At DIVs 14–16, primary cortical neurons were washed once with PBS and collected, using a cell scraper, in RIPA buffer [in mM: 50 Tris, 150 NaCl; 1% Triton X-100, 0.5% deoxycholic acid, 0.1% SDS, protease and phosphatase inhibitor cocktails (Roche), pH 7.5]. Cells lysates were incubated on ice for 30 min, centrifuged at 13,000× *g* for 15 min at 4 °C and the supernatant was collected. Proteins concentration was measured using a BCA assay kit (EuroClone, Pero, Milan, Italy). In total, 40 μg of protein was loaded onto polyacrylamide gels (8, 12, 15%) and immunoblotted as previously described [25]. After overnight primary antibody incubation, secondary species-specific horseradish peroxidase (HRP)-coupled antibodies (BioRad) were used.

The proteins were visualized by the chemiluminescent reagent ECL (Amersham, GE Healthcare, Chicago, IL, USA) or LiteAblot TURBO (EuroClone, Pero, Milan, Italy) on an Uvitec Mini HD9 (Eppendorf, Milan, Italy) apparatus. The intensity of the bands was analyzed using the ImageJ 1.3 (National Institutes of Health) software program.

Actin, tubulin and heat shock protein 90 (HSP90) protein levels were used as loading controls. Isotype-matched, HRP-conjugated secondary antibodies (Bio-Rad, Hercules, CA, USA) were used for detection (GE Healthcare). Different housekeeping proteins have been used as loading controls in order to find a molecule with a suitable molecular weight for each blot, depending on the target protein detected on the specific membrane.

### 2.7. TMRM Experiments

Mitochondrial membrane potential (ΔΨm) was detected using the fluorescent dye tetramethyl rhodamine methyl ester (TMRM).

Primary cortical neurons (DIVs 14–16) were incubated for 30 min at 37 °C in mKRB supplemented with 1 mM CaCl_2_ or in a modified experimental saline that includes 10 mM NaCl and 130 mM K^+^-gluconate in the absence of CaCl_2_. K^+^-gluconate dissipates the plasma membrane (PM) potential (ΔV), minimizing the contribution of ΔV to the TMRM signal [26,27]. Two different protocols of TMRM loading have been used: (1) 10 nM TMRM was used in mKRB supplemented with 1 mM CaCl_2_ supplemented 2 μg/mL cyclosporin H (CsH) to inhibit multidrug-resistance pumps, which could affect TMRM loading; (2) 20 nM TMRM (always in the presence of 2 μg/mL CsH) was used to compensate for the K^+^-gluconate induced loss of ΔV.

TMRM fluorescence was recorded using 40× objective (SFluor 40× N.A. 1.3, Nikon, Minato, Tokyo, Japan) on an inverted microscope (Nikon Ti-E). Fluorescence illumination used a 50–75 W Lamp (USHIO UXLS50A) and 550 nm excitation wavelengths was obtained using a monochromator (Optoscan CAIRN-Research, Faversham, UK) controlled by NIS-ELEMENTS AR (Nikon) software. The emitted fluorescence was collected using an FF-570-Di01Dichroic (Semrock, Lake Forest, IL, USA) and a 620/52 nm (Semrock) filter. Images were acquired every 60 s, with 50 ms exposure times at each wavelength, by a Zyla-CMOS 4.2-P (Andor, Oxford Instruments, Belfast, Northern Ireland). Where indicated, rotenone (1 μM) or antimycin-A (1 μM) were added. At the end of each experiment, 10 μM FCCP was added to assess the correct distribution of the dye. Images were exported as TIFF, and the background was subtracted and analyzed with ImageJ (National Institutes of Health). Data are presented as F%, meaning that fluorescence values have been normalized to final (after FCCP addition) and initial (basal) fluorescence. Pseudocolor images have been generated using ImageJ, employing the lookup table function “spectrum”.

### 2.8. Immunofluorescence (IF) and Confocal Analysis

Cortical neurons (DIVs 14–16) infected with AAV-hSyn-4mtD3mCerulean3+16 or AAV9-hSyn-split-GFP-based contact site sensor (SPLICS_s_) were first washed with PBS (once) and then fixed in 4% PFA in PBS supplemented with 0.1 g/mL sucrose. After 15 min in PFA, cells were washed 3 times (3 × 5 min) in PBS and then quenched 20 min with NH_4_Cl (50 mM in PBS). Non-infected neurons were stained for cytochrome *c* for mitochondrial morphology analysis. After the fixation and quenching step, they were permeabilized for 3 min with 0.1% Triton X-100 in PBS and blocked with a PBS solution containing 2% BSA, 10% goat serum and 0.2% gelatin for 30 min. Cells were incubated with primary antibodies diluted in blocking solution (dilution for cytochrome *c* 1:200) for 1.5 h at RT and washed three times (3 × 5 min) with the blocking solution. Cells were then incubated for 45 min at RT with AlexaFluor 555-conjugated secondary antibodies (Life Technologies; 1:300 dilution in blocking solution). Coverslips were washed 3 times (3 × 5 min) with the blocking solution and then with PBS (10 min); finally, they were mounted with Mowiol.

Images were collected on a Leica TCS SP5 II confocal system using a WLL white laser (Leica), equipped with a PlanApo 100× (numerical aperture 1.4 objective). For all images, the pinhole was set to 1 Airy unit. Confocal microscopy imaging was performed at 1024 × 1024 pixels per image, with a 0.2 Hz acquisition rate. Each channel was collected independently, and photomultiplier gain was adjusted and maintained among different experiments to minimize background and avoid saturation. Once acquired, images were background subtracted and not modified further before analysis. Images were elaborated with ImageJ (National Institutes of Health).

*Mitochondrial-ER colocalization:* To count ER–mitochondria contacts, a complete z-stack of the cell was acquired every 0.42 µm. Z-stacks were processed using Fiji. Images were first convolved and filtered using the Gaussian Blur filter. A 3D reconstruction of the resulting image was obtained using the Volume J plugin. Two selected faces of the 3D rendering were then thresholded and used to count ER–mitochondria contact sites.

*Mitochondrial morphology:* Mitochondria labelled with anti-cytochrome *c* antibody and neuronal staining obtained with NeuroTrace™ 640/660 (Invitrogen, ThermoFisher Scientific) or expressing the 4mtD3mCerulean3+16 were morphologically analyzed as described in [28] using the Fiji software [29]. Briefly, the background was subtracted, and a convolve filter and a mask were applied. Using the software’s analyze particle function, the circularity, the roundness, the aspect ratio (AR) and perimeter of mitochondria were calculated to assess mitochondrial morphology. Results are expressed as the average of values per cell profile.

### 2.9. Nonyl Acridine Orange (NAO) Staining

NAO staining has been used to measure mitochondrial content in cells. The dye binds specifically to cardiolipin in mitochondria. Neurons were seeded in a 6-well-plate and transfected as detailed above. NAO dye (400 nM, Sigma Aldrich, St. Louis, MO, USA) was added to cells at 14–16 DIVs in mKRB. Cells were stained for 30 min at 37 °C, washed with mKRB, detached mechanically by a soft scraping and suspended in mKRB at the concentration of 100,000 cells/mL. Mitochondrial mass was assessed by flow cytometry using the FACS Canto II flow cytometer (Becton Dickinson, Franklin Lakes, NJ, USA).

### 2.10. Drugs

Drugs used: cyclopiazonic acid (CPA, Abcam biochemical ab120300); oligomycin-A (Sigma Aldrich); FCCP (Sigma Aldrich, C2920); rotenone (Sigma Aldrich, R8875); antimycin-A (Sigma Aldrich, A8674); glutamate (Sigma Aldrich, G1251); ATP (Sigma Aldrich, A2383); carbachol (Sigma Aldrich, C4382); picrotoxin (Sigma Aldrich, P1675).

### 2.11. Antibodies

Antibodies used: anti-PS1 (Calbiochem, 529592); anti-cytochrome *c* (Cell signaling Technology, 12963, Danvers, MA, USA); anti-hexokinase 1 (HK1,ThermoFisher Scientific, #MA5-14789); anti-mitochondria pyruvate carrier 1 (MPC1, D2L9I, Cell signaling Technology, 14462); anti-mitochondria pyruvate carrier 2 (MPC2, D4I7G, Cell signaling, Technology 46141); anti-OXPHOS (Oxidative Phosphorylation) MitoProfile (MS604, MitoSciences, Eugene, OR, USA); anti-tubulin (Santa Cruz Biotech, Dallas, TX, USA, sc-53646); anti-actin (Sigma-Aldrich, A4700); anti-Lactate Dehydrogenase A (LDHA, Novus Biologicals, NBP1-48336, Milan, Italy); anti-HSP90 (BD Bioscience, BD610418, San Jose, CA, USA); anti-Glucose transporter 3 (Glut3, Abcam, EPR20508, Cambridge, UK); anti-cyclophilin D (Abcam, ab11024); anti-Mitofusin2 (MFN2, Abcam, ab50838); anti-Mitofusin1 (MFN1, Santa Cruz Biotech, sc166644); anti-Translocase Of Outer Mitochondrial Membrane 20 (TOM20, Santa Cruz Biotech, FL-145); anti-mitochondrial Ca^2+^ uniporter (MCU, HPA016480, Sigma-Aldrich); anti-mitochondrial Na^+^/Ca^2+^ (NCLX, Invitrogen, PA5-101901).

### 2.12. Statistical Analyses

All data are representative of at least 3 different cultures. Data were analyzed using Origin 7.5 SR5 (OriginLab Corporation, Northampton, Massachusetts, USA) and ImageJ (National Institutes of Health). Significance was calculated by unpaired Student’s *t*-test for normally distributed data or Wilcoxon rank-sum test for data not following normal distribution (* = *p* < 0.05, **=*p* < 0.01, *** = *p* < 0.001). Unless otherwise specified, numerical values presented throughout the text refer to mean ± SEM.

## 3. Results

### 3.1. Ca^2+^ Handling in PS2–/– Neurons

#### 3.1.1. Cytosolic Ca^2+^ Handling in PS2–/– Neurons

Cytosolic Ca^2+^ responses were evaluated in primary cortical neuronal cultures from WT and PS2–/– newborn mice. Since PS1 and PS2 can have differential effects on cell Ca^2+^ handling (revised in [30]), and a coregulation of PS1 and PS2 expressions has been previously reported [5,31], we firstly assessed PS1 expression level in WT and PS2–/– cortical neurons finding a slight increase, although nonsignificant, in this protein in the absence of PS2 (Figure S1A).

At 7 DIVs, neurons were transduced with an AAV9 (see Materials and Methods), carrying a newly generated cytosolic FRET-based Ca^2+^ probe, the D3mCerulean3+16 [32], under the control of the human-synapsin (hSyn) [33] promoter, to express the indicator exclusively in neurons.

**Figure 1:**
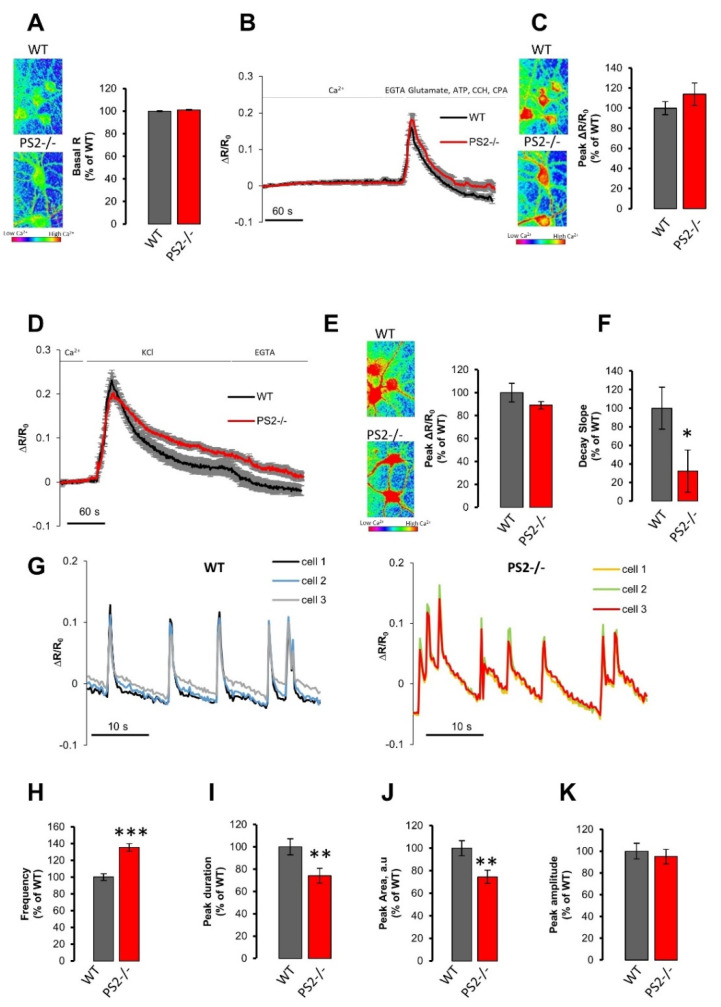
The lack of presenilin 2 (PS2) does not markedly alter the cytosolic Ca^2+^ handling, but induces neuronal hyperexcitability. (**A**) *Left.* The cytosolic resting [Ca^2+^] is visualized employing a pseudocolored scale that starts from blue-purple, low [Ca^2+^], and turns to yellow-red, high [Ca^2+^], as shown by the calibration bar. *Right.* The bar graph shows the mean basal R ± SEM, as a percentage of wild-type (WT), obtained in WT (gray, *n* = 67 cells of 10 independent cultures) or PS2–/– (red, *n* = 104 cells of 9 independent cultures) cortical neurons. (**B**) Average traces of cytosolic Ca^2+^ kinetics in WT (black) or PS2–/– (red) cortical neurons expressing the cytosolic Ca^2+^ probe D3mCerulean3+16, bathed in mKRB and exposed to a mix of drugs [500 µM carbachol (CCH), 100 µM glutamate, 100 µM ATP and 20 μM cyclopiazonic acid (CPA)], after 20 s of EGTA (0.6 mM)-containing mKRB perfusion. Data are shown as mean ± SEM of ΔR⁄R_0_ obtained in WT (*n* = 31 cells of 4 independent cultures) or PS2–/– (*n* = 35 cells of 3 independent cultures) cortical neurons. (**C**) *Left.* The cytosolic Ca^2+^ peak is visualized as described in panel A. *Right.* The bar graph shows the mean ± SEM of cytosolic Ca^2+^ peak amplitude, as a percentage of WT, obtained in WT (gray) or PS2–/– (red) cortical neurons. (**D**) Average traces of cytosolic Ca^2+^ kinetics in WT (black) or PS2 /- (red) cortical neurons expressing the cytosolic Ca^2+^ probe D3mCerulean3+16, bathed in mKRB and then perfused in KCl (30 mM)-containing iso-osmotic saline. Data are shown as mean ± SEM of ΔR⁄R_0_ obtained in WT (*n* = 36 cells of 6 independent cultures) or PS2–/– (*n*= 69 cells of 6 independent cultures) cortical neurons. (**E,F**) The bar graph shows, as a percentage of WT, obtained in WT (gray) or PS2–/– (red) cortical neurons, the mean ± SEM of (**E**) cytosolic Ca^2+^ peak amplitude and of (**F**) the recovery phase slope (calculated as linear fit of the first 30 s of the decay phase). * *p* < 0.05, The cytosolic Ca^2+^ peak is visualized in panel E *Left* as described in panel A. (**G**) Representative traces of cytosolic Ca^2+^ kinetics in WT (left) or PS2–/– (right) cortical neurons expressing the cytosolic Ca^2+^ probe D3mCerulean3+16 and stimulated by picrotoxin (25 µM) application in mKRB. The bar graph shows the mean ± SEM of (**H**) frequency, calculated as average number of Ca^2+^ spikes per minutes, *** *p* < 0.001; (**I**) peak duration, ** *p* < 0.01; (**J**) peak area (arbitrary units, a.u.), ** *p* < 0.01; (**K**) peak amplitude, as a percentage of WT, obtained in WT (gray, *n* = 132 cells of 5 independent cultures) or PS2–/– (red, *n* = 131 cells of 5 independent cultures) cortical neurons challenged with picrotoxin.

Resting cytosolic Ca^2+^ levels in cortical neurons (used at 14–16 DIVs) were indistinguishable in the two genotypes (Figure 1A), as reported in FAD-PS2-expressing neurons [34]. Neurons were then challenged, in the absence of external Ca^2+^ (to avoid interference from Ca^2+^ influx), with a mixture of metabotropic stimuli in the presence of a SERCA pump blocker (see legend of Figure 1 and Materials and Methods). The obtained cytosolic Ca^2+^ rise was again unchanged between the two genotypes (Figure 1B,C). Similarly, in PS2–/– hippocampal neurons, the resting cytosolic Ca^2+^ level (Figure S1B) and the cytosolic Ca^2+^ rise elicited by Ca^2+^ mobilization from the stores (Figure S1C,D) do not show any significant differences compared to those observed in WT cells. This phenotype differs from that observed in FAD-PS2-expressing neurons, characterized by a marked reduction in this parameter [12].

Furthermore, the cytosolic Ca^2+^ increase in response to a depolarizing stimulus (KCl) was also very similar in PS2–/– and control cells (Figure 1D,E), as previously reported in neurons from FAD-PS2 transgenic (tg) mice [12]. It is noteworthy, however, that the recovery phase of the KCl-induced cytosolic Ca^2+^ increase was significantly slower in PS2–/– cortical neurons, compared to that in WT cells (Figure 1F). This feature could be due to multiple reasons, such as diverse expression/modulation of voltage-operated Ca^2+^ channels (VOCCs), differences in the PM ΔV, in the level of Ca^2+^ buffering proteins or in the Ca^2+^ efflux pathways. Further investigations will be needed to clarify this issue. Of note, this alteration in cytosolic Ca^2+^ handling emerges in PS2–/– neurons only when a considerable amount of Ca^2+^ enters the cell, as elicited by a depolarizing stimulus, while it is not observed upon metabotropic cell stimulations.

#### 3.1.2. Neuronal Hyperexcitability in PS2–/– Neurons

Another FAD- and SAD-related phenotype is the neuronal hyperexcitability [35,36,37]. We previously demonstrated that, in cortical neurons and hippocampal slices from FAD-PS2 tg mice, B6.152H (double tg PS2N141I/APPSwe [38]) and PS2.30H (single tg PS2N141I [39]) mice, the frequency of neuronal Ca^2+^ spikes, induced by picrotoxin application, were increased, compared to those present in WT animals, without differences in the peak amplitude of each spike [12].

Because spontaneous Ca^2+^ activity is rare in cultured neurons, we exploited the same protocol to investigate neuronal excitability in PS2–/– cortical neurons. The addition of picrotoxin, by inhibiting GABA-A receptors, induced synchronous Ca^2+^ spikes in cortical neurons (Figure 1G), that were increased in frequency (Figure 1H) and reduced in peak duration (Figure 1I) and area (Figure 1J) in PS2–/– cells, compared to WT, without any effect on the peak amplitude (Figure 1K), thus resembling the hyperexcitability phenotype previously observed in cortical neurons from B6.152H and PS2.30H mice [12]. As previously shown, picrotoxin-induced cytosolic Ca^2+^ spikes were generated by glutamate-dependent activation of AMPA and NMDA receptors, upon the removal of the inhibitory action of GABA-A receptors. The overlapping picrotoxin-dependent overlapping phenotype found in FAD-PS2-expressing [12] and PS2–/– neurons (this work) might indicate an inhibitory role of endogenous PS2 with regard to the glutamate receptors. This function could be lost when PS2 is mutated. Further in-depth studies are, however, necessary to demonstrate this hypothesis.

#### 3.1.3. Mitochondrial Ca^2+^ Handling in PS2–/– Neurons

We have previously demonstrated that FAD-PS2 mutations cause a reduction in mitochondrial Ca^2+^ uptake in response to inositol trisphosphate (IP_3_)-generating stimuli because of the lower ER Ca^2+^ content in cells expressing the PS2 mutants [11,12,13]. On the other hand, the expression of the same FAD-PS2 mutants induces an increase in the physical apposition between ER and mitochondria [11,12,13,17,40], potentiating organelle Ca^2+^ cross-talk. Indeed, FAD-PS2 has been shown to be enriched at sites of close proximity between ER and mitochondria, at specific ER membrane domains, called mitochondria-associated membranes (MAMs) [13,41].

We thus tested cortical neurons from WT and PS2–/– mice for their mitochondrial Ca^2+^ responses by infecting them, at 7 DIV, with an AAV9 carrying a newly generated mitochondrial FRET-based Cameleon probe, the 4mtD3mCerulean3+16 [32], expressed under the control of the hSyn promoter. As for the cytosol, mitochondrial Ca^2+^ experiments were performed at 14–16 DIVs.

**Figure 2:**
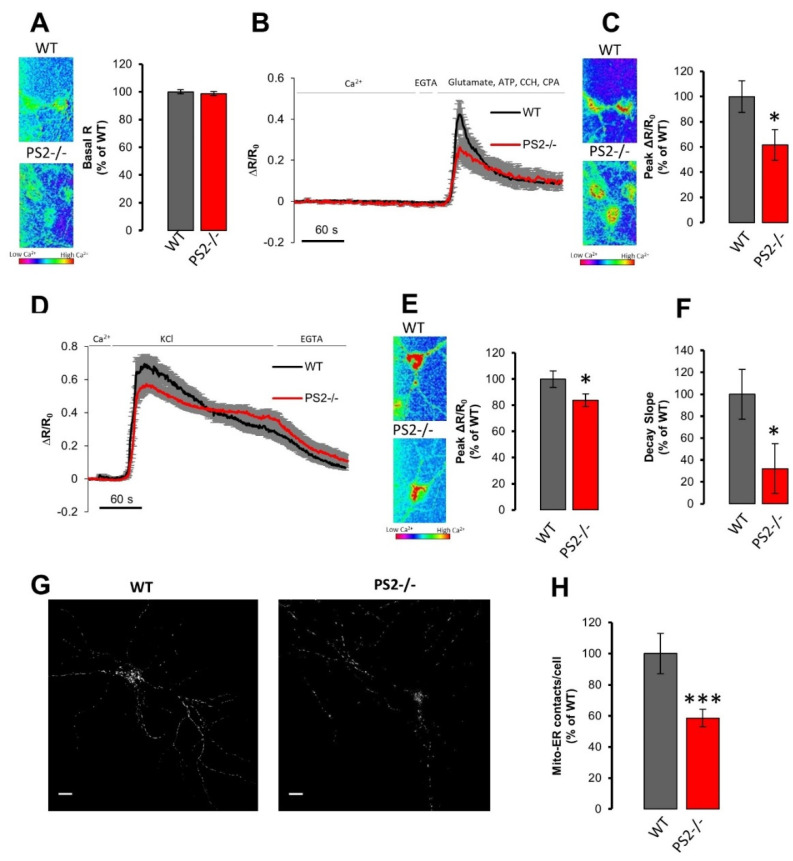
The lack of PS2 dampens mitochondrial Ca^2+^ uptake and reduces endoplasmic reticulum (ER)–mitochondria contact sites. (**A**) *Left.* The mitochondria resting [Ca^2+^] is visualized employing a pseudocolored scale as described in Figure 1A *Right.* The bar graph shows the mean basal R ± SEM of in WT (gray) or PS2–/– (red) cortical neurons expressing the mitochondrial Ca^2+^ probe 4mtD3mCerulean3+16, as a percentage of WT, obtained in WT (gray, *n* = 61 cells of 8 independent cultures) or PS2–/– (red, *n* = 72 cells of 8 independent cultures) cortical neurons. (**B**) Average traces of mitochondrial Ca^2+^ kinetics in WT (black) or PS2–/– (red) cortical neurons expressing the mitochondrial Ca^2+^ probe 4mtD3mCerulean3+16, bathed in mKRB and exposed to the mix of drugs described in Figure 1B, after 20 s of EGTA (0.6 mM)-containing mKRB perfusion. Data are shown as mean ± SEM of ΔR⁄R_0_ obtained in WT (*n* = 26 cells of 3 independent cultures) or PS2–/– (*n* = 19 cells of 3 independent cultures) cortical neurons. (**C**) *Left.* The mitochondria Ca^2+^ peak is visualized as described in Figure 1A. *Right.* The bar graph shows mean ± SEM of mitochondrial Ca^2+^ peak amplitude, as a percentage of WT, obtained in WT (gray) or PS2–/– (red) cortical neurons. * *p* < 0.05 (**D**) Average traces of mitochondrial Ca^2+^ kinetics in WT (black) or PS2–/– (red) cortical neurons expressing the mitochondria Ca^2+^ probe 4mtD3mCerulean3+16, bathed in mKRB and then perfused in KCl (30 mM)-containing iso-osmotic saline. Data are shown as mean ± SEM of ΔR⁄R_0_ obtained in WT (*n* = 35 cells of 5 independent cultures) or PS2–/– (*n* = 34 cells of 5 independent cultures) cortical neurons. (**E,F**) The bar graph shows, as a percentage of WT, obtained in WT (gray) or PS2–/– (red) cortical neurons, the mean ± SEM of (**E**) the mitochondrial Ca^2+^ peak amplitude (* *p* < 0.05) and of (**F**) the recovery phase slope (calculated by linear fit of the first 30 s of the decay phase, * *p* < 0.05). The mitochondria Ca^2+^ peak is visualized in panel E left as described in in Figure 1A. (**G**) Representative confocal images of WT (left) and PS2–/– (right) neurons infected with Adeno-Associated Virus (AAV)-human-synapsin (hSyn)-split-GFP-based contact site sensor (SPLICS_s_) probe_._ Scale bar 10 µm. (**H**) The bar graph shows the mean number of dots ± SEM obtained in WT (gray, *n* = 33 cells of 3 independent cultures) or PS2–/– (red, *n* = 34 cells of 3 independent cultures) cortical neurons (as a percentage of WT), *** *p* < 0.001.

No difference between WT and PS2–/– cortical neurons was observed in the resting mitochondrial Ca^2+^ levels (Figure 2A). Since the *K*_d_ for Ca^2+^ of the used probe is 6.5 μM [32] and Ca^2+^ concentration ([Ca^2+^]) in the mitochondrial matrix at rest is in the nm range, another mitochondrial genetically encoded Ca^2+^ indicator (GECI) with higher Ca^2+^ affinity, 4mtGCaMP6f [42,43], was also used to measure resting mitochondrial [Ca^2+^], confirming our results (Figure S2A).

Neurons were then stimulated with the mixture of metabotropic stimuli used before (Figure 1), in the presence of a SERCA pump blocker and in the absence of external Ca^2+^ [12]. A significant reduction in mitochondrial Ca^2+^ peaks (Figure 2B,C) was observed in PS2–/– neurons, compared to WT cells, although the cytosolic response to the same stimulation was indistinguishable (Figure 1B,C). Similar results were also observed in PS2–/– hippocampal neurons (Figure S2B–D). It is noteworthy that the lack of PS2 and the expression of FAD-PS2 mutants have similar overall effects on mitochondrial Ca^2+^ handling in response to Ca^2+^ mobilization from the ER [11,12,13,17]. However, in PS2–/– neurons, cytosolic Ca^2+^ peaks observed upon ER Ca^2+^ release were unaffected with respect to control cells (Figure 1B,C and Figure S1C–D), while those observed in FAD-PS2-expressing cells were significantly reduced [7,8,9,10,11,12,13,14,15].

To investigate whether the observed defective mitochondrial Ca^2+^ response in PS2–/– neurons was caused by a reduced ER to mitochondria Ca^2+^ shuttling (due, for example, to an increased distance between the two organelles) and/or by an overall mitochondrial impairment, cortical neurons were stimulated with KCl (30 mM) to induce a large Ca^2+^ influx across the PM. Mitochondrial Ca^2+^ uptake was also slightly reduced in PS2–/– neurons upon this stimulation (Figure 2D,E), although to a lesser extent when compared to the metabotropic stimulation protocol (Figure 2B,C and Figure S2C,D). Of note, the rate of Ca^2+^ efflux due to the KCl stimulation was slower in PS2–/– cells, compared to that in controls (Figure 2F), resembling what was observed in the cytosol (Figure 1F).

Overall, these results suggest that the KO of PS2 causes an impairment in mitochondrial Ca^2+^ handling, as was the case with the expression of FAD-PS2 mutants [11,12,13,17], although by mechanisms not related to the content of the intracellular Ca^2+^ stores.

#### 3.1.4. ER–Mitochondria Apposition in PS2–/– Neurons

As mentioned above, an increased ER–mitochondria proximity was found in cell lines, neurons and patient-derived fibroblasts carrying different FAD-PS2 mutations [11,12,13,40]. An increased (or decreased) tethering between the two organelles could allow the formation of more (or less) microdomains of high [Ca^2+^], the so-called Ca^2+^ hotspots, on the mitochondrial surface, close to the mouth of ER-located Ca^2+^-releasing channels, thus favoring (or reducing) mitochondrial Ca^2+^ uptake for similar IP_3_-generated cytosolic Ca^2+^ rises [11,44,45,46].

To investigate whether ER–mitochondria proximity was modified in PS2–/– neurons, and thus involved in the defective mitochondrial Ca^2+^ phenotype observed in these cells, we took advantage of a recently generated probe, called the split-GFP-based contact site sensor (SPLICS [40]). This probe allows the detection of organelle contact sites, exploiting the split-GFP approach. By this methodology, it has been shown that patient-derived fibroblasts carrying the FAD-PS2-NI141I mutation presented double the number of contact sites between ER and mitochondria compared to control cells [40], confirming the higher organelle proximity observed previously when using other methods in the same fibroblasts [11,13] and in neurons from both B6.152H and PS2.30H mice [12]. WT and PS2–/– cortical neurons were infected with specific AAV9 to express the SPLICS probe under the control of the hSyn promoter (Figure 2G). Confocal z-stack images of WT and PS2–/– neurons showed ER–mitochondria contact sites as fluorescent dots (Figure 2G), that were significantly decreased in number in PS2–/– neurons compared to WT cells (Figure 2H), according to what we previously showed in SH-SY5Y cells in which PS2 protein level was reduced by a specific siRNA [11].

Thus, PS2–/– neurons show a marked decrease in ER–mitochondria contact sites, the opposite of what is consistently observed in FAD-PS2 mutant-expressing cells [11,12,13,40].

### 3.2. Mitochondrial Functionality in PS2–/– Neurons

#### 3.2.1. Mitochondrial Morphology and Mass in PS2–/– Neurons

Mitochondrial morphology was assessed by staining the mitochondrial network with an anti-cytochrome *c* antibody in cells identified as neurons by NeuroTrace™ (Figure S3A). No significant differences were found between controls and PS2–/– neurons in regard to mitochondrial morphology and organelle numbers (Figure S3A–E). Similar results were obtained in neurons infected with AAV-hSyn-4mtD3mCeruelan3+16 (Figure S3F–J), indicating that the infection procedure (and the expression of an exogenous mitochondrial protein) does not modify the organelle network.

**Figure 3:**
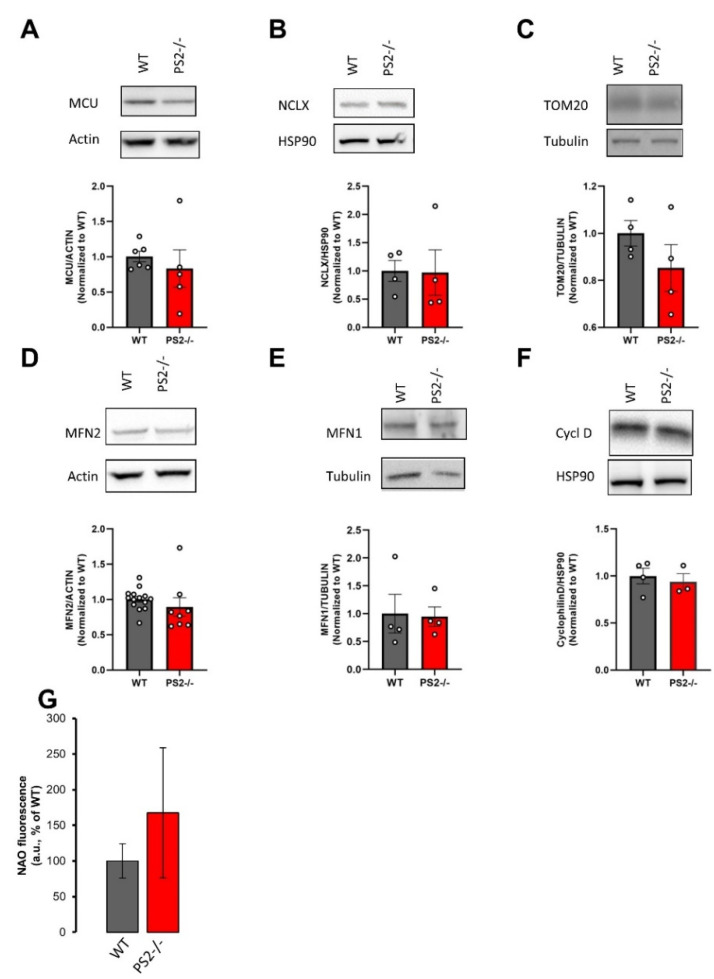
The lack of PS2 does not affect the mitochondrial mass. Representative Western blots of different mitochondrial protein levels in WT and PS2–/– cortical neurons. Each sample was run in duplicate. The corresponding scatter plot represents the mean values ± SEM. Values were first normalized to their internal housekeeping proteins (as indicated) and then to those of WT samples. (**A**–**G**) Representative Western blot and quantification of: (**A**) mitochondrial Ca^2+^ uniporter (MCU) protein levels of 6 independent cultures from WT mice and 5 independent cultures from PS2–/– mice; (**B**) NCLX protein levels of 4 independent cultures from WT mice and from PS2–/– mice; (**C**) TOM20 protein levels of 4 independent cultures from WT mice and from PS2–/– mice; (**D**) MFN2 protein levels of 14 independent cultures from WT mice and 8 independent cultures from PS2–/– mice; (**E**) MFN1 protein levels of 4 independent cultures from WT mice and from PS2–/– mice; (**F**) cyclophilin D (cycl D) protein levels of 4 independent cultures from WT mice and 3 independent cultures from PS2–/– mice. (**G**) The bar graph shows the mean values ± SEM of Nonyl Acridine Orange (NAO) fluorescence (arbitrary units) from 4 independent cultures from WT mice and 3 independent cultures from PS2–/– mice, as a percentage of WT.

Furthermore, no significant differences were found in the expression levels of different proteins located at either the inner mitochondrial membrane (IMM), e.g., the mitochondrial Ca^2+^ uniporter (MCU, Figure 3A) and the mitochondrial Na^+^/Ca^2+^ exchanger (NCLX, Figure 3B), the outer mitochondrial membrane (OMM), e.g., the translocase of the outer membrane 20 (TOM-20, Figure 3C), mitofusin 2 (MFN2, Figure 3D) and mitofusin 1 (MFN1, Figure 3E), or the mitochondrial matrix, e.g., cyclophilin-D (Cycl D, Figure 3F).

Moreover, mitochondrial mass was further estimated by cardiolipin staining combined with flow cytometry quantification [17]. As shown in Figure 3G, no significant differences in this parameter were detected between WT and PS2–/– cortical neurons.

Taken together, these results indicate that neither mitochondrial mass nor organelle morphology are modified by the lack of PS2 expression.

#### 3.2.2. Mitochondrial Membrane Potential

We have previously shown that mitochondria of hippocampal neurons from PS2.30H mice depolarize faster upon complex I inhibition [17], and mitochondria of cortical neurons from B6.152H mice undergo a stronger depolarization upon the application of low concentrations of the uncoupler FCCP [16], suggesting a mitochondrial dysfunction in neurons from both FAD-PS2 tg animals.

Since mitochondrial Ca^2+^ uptake strongly depends on organelle membrane potential (ΔΨm), the effect of PS2 absence on organelle polarization was investigated by TMRM, a cell-permeant cationic dye that accumulates inside the mitochondrial matrix as a function of ΔΨm.

**Figure 4:**
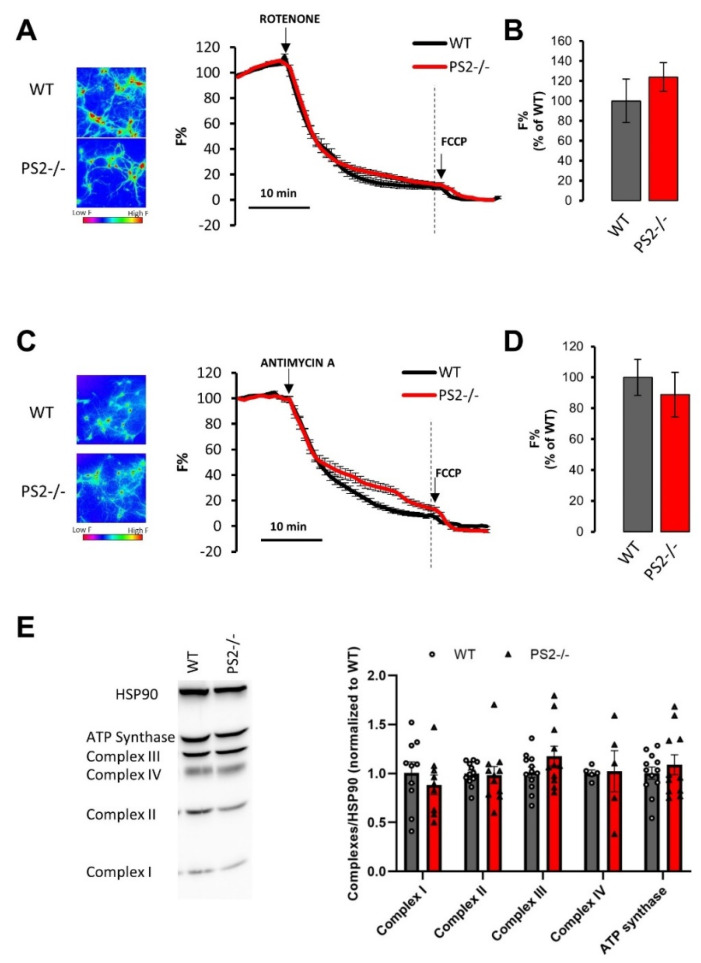
The lack of PS2 does not affect ΔΨm and the expression levels of the different respiratory chain complexes. (**A**–**C**) Average traces of WT (black) or PS2–/– (red) cortical neurons loaded with tetramethyl rhodamine methyl ester (TMRM), bathed in mKRB and exposed to rotenone (1 µm, panel **A**) or antimycin A (1 µm, panel **C**) for 30 min and carbonyl cyanide-4-(trifluoromethoxy)phenylhydrazone (FCCP) (10 µm) for 10 min. Data are shown as mean ± SEM of F% obtained in WT (rotenone: *n* = 59 cells of 5 independent cultures; antimycin A: *n* = 117 cells of 5 independent cultures) or PS2–/– (rotenone: *n* = 85 cells of 5 independent cultures; antimycin A: *n* = 67 cells of 5 independent cultures) cortical neurons. The TMRM fluorescence, at rest, was visualized by employing a pseudocolored scale that starts from blue-purple, low TMRM accumulation, and turns to yellow-red, high TMRM accumulation (as shown by the calibration bar), in panels A and C (left part). (**B**–**D**) The bar graph shows the mean ± SEM of F%, as a percentage of WT, obtained in WT (gray) or PS2–/– (red) cortical neurons upon rotenone (**B**) or antimycin A (**D**) addition, calculated at the time point indicated by the dashed line of panel (**A**) or (**C**), respectively. (**E**) Representative Western blot of respiratory chain complexes and ATP synthase levels in WT and PS2–/– neurons. The scatter plots show the mean values ± SEM of 9 independent cultures for WT and 7 independent cultures for PS2–/–, run in duplicate. Values were first normalized by their internal HSP90 levels and then to those of WT samples.

Neurons were first challenged with rotenone, a specific inhibitor of complex I (Figure 4A), and no difference between PS2–/– and WT cells was observed in the TMRM fluorescence decay (Figure 4B). Antimycin A, a complex III blocker, was then used (Figure 4C), and the mitochondrial depolarization in the PS2–/– genotype was not significantly different to controls (Figure 4D).

Since TMRM accumulation within mitochondria depends on dye concentration in the cytosol, and thus on the PM ΔV, to exclude a possible contribution of ΔV, TMRM measurements were also performed in a K^+^-gluconate-containing medium. Under these experimental setting, ΔV is close to 0. Additionally, in this condition, no differences in TMRM fluorescence kinetics were observed in response to rotenone (Figure S4A,B) or antimycin A (Figure S4C,D) between PS2–/– neurons and controls.

Finally, the expression level of the respiratory chain complexes and of ATP synthase were indistinguishable between PS2–/– and WT neurons (Figure 4E), as also reported for cortical neurons from PS2.30H [17] and B6.152H [16] mice.

#### 3.2.3. Mitochondrial Respiration, Glycolysis and ATP Production

We have previously shown that the maximal oxygen consumption rate (OCR) of hippocampal neurons from PS2.30H mice is lower compared to that of WT cells [17]. Similar results have been obtained in cortical neurons from B6.152H mice. In this latter FAD-PS2 mouse model, a reduced spare capacity and cell respiratory control ratio have been also described [16].

**Figure 5:**
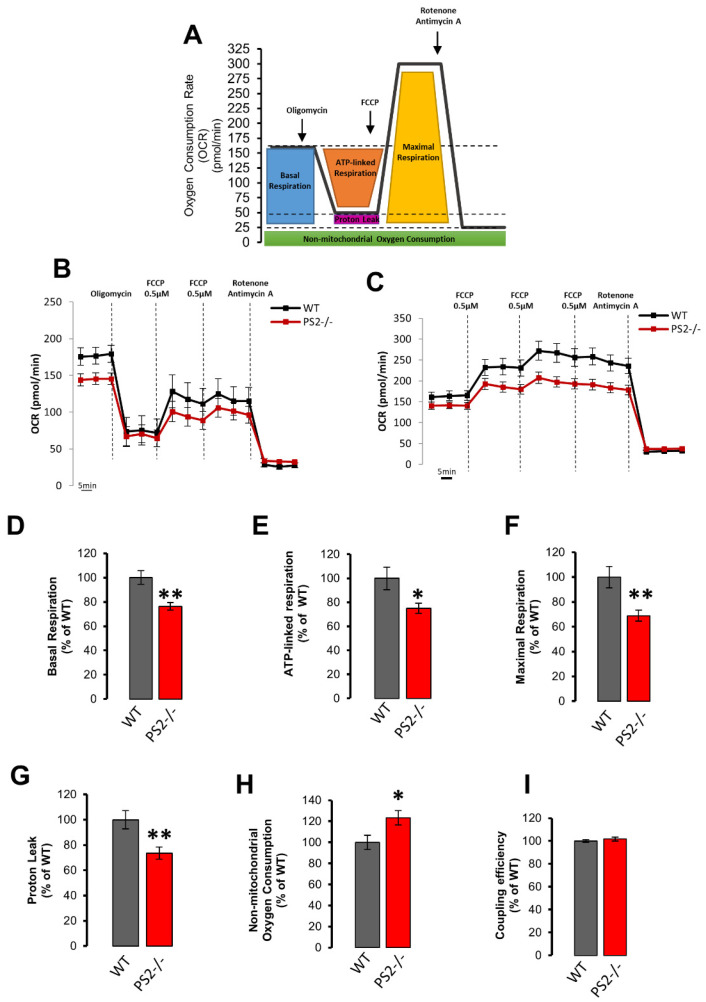
The lack of PS2 slightly affects mitochondrial respiration. (**A**) Oxygen consumption rate (OCR) experimental protocol. (**B**–**C**) OCR mean traces in WT (black) and PS2–/– (red) primary cortical neurons. Oligomycin (Oligo, 1 μM), FCCP (0.5 μM), and antimycin A (0.1 μM) plus rotenone (1 μM) were added as indicated. Data are plotted as mean pmol/min ± SEM (*n* > 45 wells from at least 6 independent cultures). (**D**–**I**) Quantification of OCR measurements in panels B and C. The bar graph shows the mean ± SEM as a percentage of WT, obtained in WT (gray) or PS2–/– (red) cortical neurons of: (**D**) the basal OCR (calculated as differences between initial OCR and OCR after antimycin A/rotenone addition), ** *p* < 0.01; (**E**) the mean ATP-linked OCR (calculated as differences between initial OCR and OCR after oligomycin addition), * *p* < 0.05; (**F**) the mean of the maximal respiratory capacity (calculated as difference between OCR after FCCP addition and the OCR after antimycin A/rotenone addition), ** *p* < 0.01; (**G**) the mean proton leak (calculated as differences between the oligomycin-sensitive OCR and OCR after antimycin A/rotenone addition), ** *p* < 0.01; (**H**) the mean non-mitochondrial OCR (OCR remaining after addition of all drugs), * *p* < 0.05; (**I**) the mean coupling efficiency (calculated as ratio between ATP-linked OCR and basal OCR).

Mitochondrial respiration was investigated, using the Seahorse platform, in WT and PS2–/– neurons (Figure 5A–C). To estimate basal and maximal respiration, we performed two distinct protocols (Figure 5B,C). Indeed, as already reported [16,22,47], the addition of the uncoupler FCCP after oligomycin prevented the possibility of reaching maximal OCR in neurons (Figure 5B). Thus, maximal respiration rate was assessed separately from oligomycin addition by adding increasing concentrations of FCCP (Figure 5C). PS2–/– cells, compared to controls, have a reduced basal OCR (mainly due to ATP synthesis and proton leak, Figure 5D), ATP-linked respiration (due to ATP synthase, Figure 5E), maximal OCR (upon uncoupler addition, Figure 5F) and proton leak (Figure 5G) and a slightly higher non-mitochondrial oxygen consumption (Figure 5H). However, the ratio between basal and oligomycin-resistant OCR, called coupling efficiency and equivalent to the classical “mitochondrial respiratory control”, was indistinguishable in the two cell types (Figure 5I).

**Figure 6:**
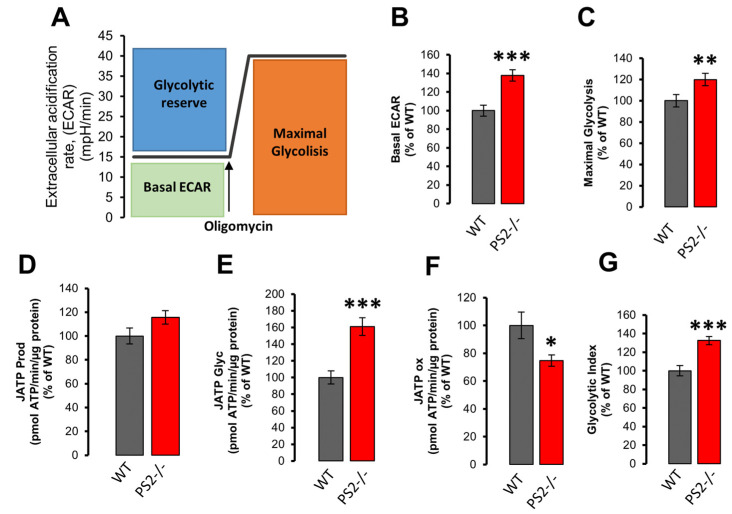
The lack of PS2 induces a metabolic switch towards glycolysis. (**A**) Extracellular acidification rate (ECAR) experimental protocol. (**B**–**E**) The bar graph shows the mean ± SEM, as a percentage of WT, obtained in WT (gray) or PS2–/– (red) cortical neurons of: (**B**) the basal ECAR, *** *p* < 0.001; (**C**) the maximal glycolysis (calculated as mean of the ECAR reached upon oligomycin addition), ** *p* < 0.01; (**D**) the total ATP production rate (JATP Prod); (**E**) the glycolytic ATP production rate (JATP Glyc), *** *p* < 0.001; (**F**) the ATP production rate due to mitochondrial respiration (OXPHOS; JATP ox), * *p* < 0.05; (**G**) the Glycolytic Index (calculated as ratio between normalized JATP Glyc and JATP), *** *p* < 0.001.

The extracellular acidification rate (ECAR, Figure 6A), an indicator of anaerobic glycolysis, was also calculated from OCR experiments (Figure 5). Increased basal ECAR (Figure 6B) and maximal glycolysis (Figure 6C) were found in PS2–/– neurons, compared to WT, while the glycolytic reserve (defined as the difference between ECAR induced by oligomycin addition and basal ECAR; Figure S5A) presented no differences between the two genotypes. According to these data, a lower OCR/ECAR ratio was calculated for neurons lacking PS2 (Figure S5B).

These results are substantially different from those obtained previously in cortical neurons from B6.152H mice, in which, compared to WT cells, a decreased basal and maximal ECAR as well as a reduced glycolytic reserve have been observed [16].

Using ECAR and OCR data, an indirect estimation of the rate of ATP production within the cell can be performed [23,24]. According to this calculation, PS2–/– neurons displayed no significant difference in total ATP production compared to WT cells (Figure 6D). However, in PS2–/– neurons, glycolytic ATP was increased (Figure 6E), while oxygen-linked ATP production appeared partially decreased (Figure 6F), suggesting a modest metabolic switch towards glycolysis in these cells (Figure 6G).

#### 3.2.4. Key Proteins in the Regulation of Glycolysis, Krebs Cycle and Oxidative Phosphorylation

To obtain some mechanistic insights into the mitochondrial phenotype found in PS2–/– neurons, the protein levels of multiple enzymes and transporters linked to mitochondrial metabolism and bioenergetics were also investigated. In particular, we measured the protein levels of: (i) hexokinase-1 (HK1), the first enzyme of glycolysis, a key protein that, by dynamically associating with the mitochondrial protein VDAC, coordinates cytosol-mitochondria metabolic cross-talk (Figure S6A); (ii) glucose transporter 3 (GLUT3), a protein specifically devoted to the transport of glucose across the PM in neurons (Figure S6B); (iii) mitochondrial pyruvate carrier (MPC), MPC1 (Figure S6C) and MPC2 subunits (Figure S6D), a protein that transports pyruvate into the mitochondrial matrix; (iv) lactate dehydrogenase (LDH), a key enzyme linking glycolysis and mitochondrial respiration (Figure S6E).

PS2–/– cortical neurons do not display differences in the total expression levels of these proteins, compared to WT, similarly to what has been reported in cortical neurons from B6.152H mice [16], whereas MPC2 has been found to be reduced in FAD-PS2-expressing cells [17].

## 4. Discussion

The recently proposed loss-of-function phenotype associated with FAD-PS1 raises a question about the possibility of extending this pathogenic hypothesis to FAD-PS2-linked cases, given the overlapping phenotype among AD patients carrying *PSEN1* or *PSEN2* mutations [5].

FAD-PS2 mutations have been associated with Ca^2+^ and metabolic dysfunctions (reviewed in [30,48,49,50]). In particular, PS2 has been implicated in the modulation of: (1) SERCA pump activity and thus ER Ca^2+^ accumulation [10]; (2) ER to mitochondria Ca^2+^ shuttling and physical organelle apposition [11,12,13,40]; (3) mitochondrial bioenergetics [16,17,51,52]. Moreover, evidence has been accumulated suggesting that mitochondrial dysfunctions are early events in AD pathogenesis, suggesting a “mitochondrial cascade hypothesis” (reviewed in [53,54,55,56]). Most of these data have been obtained in cells or tg animals overexpressing FAD-PS2, since knockin animals are not available yet. However, it is worth mentioning that we have previously demonstrated that fibroblasts from FAD patients, in which the mutated PS2 is not overexpressed resembling the pathological situation, also share the same phenotype [7,8,10,13,15,40]. Nevertheless, our finding that the effects of FAD-PS2 mutants on multiple Ca^2+^ pathways is mimicked by high expression levels of wt PS2 [7,8,11,15] might have implications for the pathogenesis of SAD, the more frequently form of the disease, where the reported decreased expression level of Repressor Element 1-Silencing Transcription factor (REST [57]) causes an increase in the endogenous PS2 expression [58].

As far as Ca^2+^ signaling is concerned, we found that PS2–/– neurons show a Ca^2+^-related phenotype different from that reported in FAD-PS2-expressing cells, except for the Ca^2+^ hyperexcitability. Indeed, PS2-linked FAD mutants have been reported to dampen ER Ca^2+^ content and mitochondrial Ca^2+^ uptake upon IP_3_-linked stimulation [7,8,9,10,11,12,13,14,15], while increasing ER–mitochondria contact sites [11,12,13,40]. Instead, PS2–/– neurons do not show alteration in ER Ca^2+^ release, whereas they display a reduced mitochondrial Ca^2+^ uptake in response to Ca^2+^ mobilization from stores, likely due to a decreased ER–mitochondria apposition. Indeed, this latter feature appears particularly evident in PS2–/– neurons, strongly supporting the idea that PS2 is a key regulator of organelle tethering [11,12,13,40].

Interestingly, when stimulated by KCl, while the peak in cytosolic Ca^2+^ is indistinguishable in the two genotypes, the decrease in the KCl-induced Ca^2+^ transient in PS2–/– cells is slower compared to that observed in control cells. This feature has not been found in FAD-PS2-expressing neurons [12]. The mechanism of this phenomenon is presently unclear but confirms that the KO of PS2 and expression of FAD-PS2 mutants have different effects on neuronal Ca^2+^ handling. The kinetics of the KCl-induced mitochondrial Ca^2+^ rise was very similar in PS2–/– and controls, although a small but significant reduction was observed in the mitochondrial Ca^2+^ peak amplitude. No significant differences, however, were observed in the expression level of the main mitochondrial Ca^2+^ handling molecular components, MCU and NCLX proteins. The simplest explanation is that, upon depolarization and activation of VOCCs, the entrance of Ca^2+^ across the PM triggers the release of Ca^2+^ from the ER, via ryanodine receptors, and the reduced apposition of mitochondria to the ER, observed in PS2–/– neurons, results in a decreased ER-to-mitochondria Ca^2+^ transfer.

Previous studies unveiled a specific role of PS2 in maintaining mitochondrial function and cell bioenergetics. Indeed, decreases in ΔΨm, reduced mitochondrial respiration, defective mitochondrial cristae morphologies and increased glycolysis have been reported in mouse embryonic fibroblasts (MEFs) lacking PS2 [51,52]. All these mitochondrial alterations were specifically due to the lack of PS2 since PS1-KO MEFs did not show any of these phenotypes [51,52]. Although, it is worth mentioning that other works reported a role of PS1 mutants in mitochondrial impairment [59,60,61,62]. Along similar lines, neurons from FAD-PS2 tg mice showed defective mitochondrial bioenergetics [16,17], according to the emerging idea of AD as a metabolic pathology, in which an energetic unbalance could strongly contribute to its genesis [53,63].

Here, we could not confirm the data reported in PS2–/– MEFs (as summarized in Table 1), as we found that PS2–/– neurons have no defects in mitochondrial morphology and number, as well as in their ΔΨm and expression levels of respiratory chain complexes and ATPase. Instead, PS2–/– neurons show a slight alteration in respiratory parameters and glycolysis. However, the coupling efficiency (i.e., the ratio between basal OCR and OCR after oligomycin) is indistinguishable in control and PS2–/– cells.

Likely, the reduced ER-to-mitochondria Ca^2+^ shuttling, due to the strong decrease in contact sites between the two organelles observed in PS2–/– neurons, could contribute to these minor defects in organelle respiration. Indeed, the constitutive, low Ca^2+^ transfer between ER and mitochondria has been shown to be important in sustaining optimal mitochondrial function and cell bioenergetics [46]. Alternative explanations are, however, possible and further investigations are thus needed to solve this point.

The PS2–/– phenotypes described here are likely due to the specific ablation of PS2, despite the presence of a slight, nonsignificant, increase in PS1 levels in these animals. Indeed, we previously demonstrated that PS1 overexpression [11,15], or downregulation [11], does not affect intracellular Ca^2+^ handling and ER–mitochondria tethering. Moreover, acute downregulation of endogenous PS2 [11] induces similar phenotypes compared to those described here on mitochondria Ca^2+^ handling and ER–mitochondria apposition by stable PS2 ablation, thus excluding any genetic compensation-related effect on these parameters. 

To our knowledge, the only work investigating the effect of PS2 ablation on mitochondrial functions has been carried out in MEF cells [51,52]. Here, we addressed this issue in primary neurons, as they represent a relevant cellular model for AD. However, since this is an in vitro study and, thus, it might have intrinsic limitations, further in vivo investigations in PS2–/– mice will be needed to extend our knowledge on the effects of PS2 ablation on brain Ca^2+^ signaling and mitochondrial function in a more physio-pathological context and during aging. Nevertheless, up to now, we have found full correspondence between in vitro and ex vivo data obtained for Ca^2+^ handling in FAD-PS2-expressing models, as shown by [12], where the results obtained in primary neurons and in acute hippocampal brain slices from FAD-PS2 transgenic mice perfectly overlap. 

Moreover, our data might have important therapeutic implications for AD. Indeed, if the hypothesis proposed by Xia et al. that FAD-PS1 mutations are loss of function was true, and this could be generally extended to FAD-PS2 mutants as well, a paradigm change in AD pathogenesis would occur, opening new therapeutic venues aimed at restoring, instead of at inhibiting, PS functions [5]. This, however, seems not to be fully confirmed, raising doubts regarding the proposed pathogenic mechanism for AD.

## 5. Conclusions

The results obtained in neurons KO for PS2 indicate that most of the cellular and molecular phenotypes described for FAD-PS2 mutations are not recapitulated by the lack of PS2 (as summarized in Table 1), but rather deletion of PS2 has mainly opposite effects compared to those induced by the expression of FAD-PS2 mutants. In particular, PS2–/– neurons have normal ER Ca^2+^ contents (while FAD-PS2 expression causes a major ER Ca^2+^ depletion) and show a strongly reduced physical apposition between ER and mitochondria (while FAD-PS2 reinforces organelle tethering, causing a large increase in these contacts). Finally, recent investigations of local field potentials in FAD-PS2 and PS2–/– mice showed that the two models substantially differ in the alterations of their brain network activity patterns [64,65], providing further evidence against the hypothesis of a loss-of-function phenotype associated with FAD-PS2 mutants.

The modest alterations in both mitochondrial Ca^2+^ handling and bioenergetics are consistent with the fact that PS2–/– mice are alive and healthy without a brain phenotype, or other defects, until 12 months of age, with the only exception of a mild pulmonary fibrosis (which is not affecting animal breathing) and the insurgence of hemorrhages with age. Moreover, a similar apoptosis rate, APP processing and Aβ production have been found in PS2–/– neurons, compared to WT cells [6]. Instead, the KO of PS1 is embryonically lethal, indicating that PS1 and PS2 overlap only partially in their functions, with PS1 being specifically indispensable for embryonic development, mainly affecting the Notch signaling pathway [66,67,68,69].

Taken together, the present data indicate that the majority of the functional effects of FAD-PS2 mutants are not related to a loss-of-function phenotype and that the ER-located protein PS2, by modulating the physical and functional coupling between ER and mitochondria, is an important regulator of cell bioenergetics.

## Figures and Tables

**Table 1:** The table summarizes the Ca^2+^ handling and mitochondrial bioenergetics parameters analyzed in PS2–/–, Familial Alzheimer’s disease (FAD)-PS2 expressing neurons and mouse embryonic fibroblasts (MEFs) PS2–/–, compared to WT. ND = not determined.

		PS2N141I	PS2N141I APPswe	MEF PS2−/−	PS2−/−(This Paper)
Cytosolic Ca^2+^ handling	Basal levels	unchanged [32]	unchanged [32]	ND	unchanged
	Store Ca^2+^ content	reduced [12]	reduced [12]	ND	unchanged
	KCl response	unchanged [12]	unchanged [12]	ND	unchanged
Neuronal hyper-excitability		increased [12]	increased [12]	ND	increased
MitochondrialCa^2+^handling	Basal levels	ND	ND	ND	unchanged
	ER–mitochondria Ca^2+^ transfer	reduced [12]	reduced [12]	ND	reduced
	KCl response	ND	ND	ND	reduced
ER–mitochondria contacts		increased [12]	increased[12]	ND	reduced
Mitochondrial morphology		unchanged	unchanged [16]	defective cristae [53]	unchanged
Mitochondrial mass		unchanged [17]	unchanged [16]	ND	unchanged
Mitochondrial membrane potential	Basal	unchanged [12]	unchanged [12, 16]	unchanged [52,53]	ND
	Complex I inhibition	faster decay [17]	unchanged [16]	ND	unchanged
	Complex III inhibition	ND	unchanged [16]	ND	unchanged
OxygenConsumptionRate (OCR)	Basal	reduced [17]	unchanged [16]	reduced [52,53]	reduced
	Maximal	reduced [17]	reduced [16]	reduced [52,53]	reduced
	ATP-linked respiration	reduced [17]	unchanged [16]	reduced [52,53]	reduced
	Proton leak	unchanged [17]	unchanged [16]	ND	reduced
	Non-mitochondrial OCR	unchanged [17]	unchanged [16]	ND	increased
Glycolysis(ECAR)	Basal	unchanged [17]	reduced [16]	increased [52,53]	increased
	Maximal Glycolysis	unchanged [17]	reduced [16]	ND	increased
Respiratory chain complexes (levels of expression)		unchanged [17]	unchanged [16]	reduced CI, CII, CIV [53]	unchanged

## Data Availability

Data is contained within the article or supplementary material.

## Supplementary Materials

The following are available online at https://www.mdpi.com/2073-4409/10/2/204/s1, Figure S1. The lack of presenilin 2 (PS2) does not alter the cytosolic Ca^2+^ handling in hippocampal neurons; Figure S2. The lack of PS2 dampens mitochondrial Ca^2+^ uptake in hippocampal neurons; Figure S3. The lack of PS2 does not affect mitochondrial morphology; Figure S4. The lack of PS2 does not affect ΔΨm; Figure S5. The lack of PS2 does not affect the glycolytic reserve, while it decreases Oxygen Consumption Rate (OCR)/Extracellular Acidification Rate (ECAR) ratio; Figure S6. The lack of PS2 does not affect proteins involved in the regulation of glycolysis, Krebs cycle and oxidative phosphorylation.
