# Supplementary material for: Calcium Signaling and Mitochondrial Function in Presenilin 2 Knock-Out Mice: Looking for Any Loss-of-Function Phenotype Related to Alzheimer’s Disease
Source: Cells. 2021 Jan 21;10(2):204. doi: 10.3390/cells10020204 (PMC7909802; doi:10.3390/cells10020204)
Supplement: Supplementary file 1 [file cells-10-00204-s001.pdf]

Supplementary Material

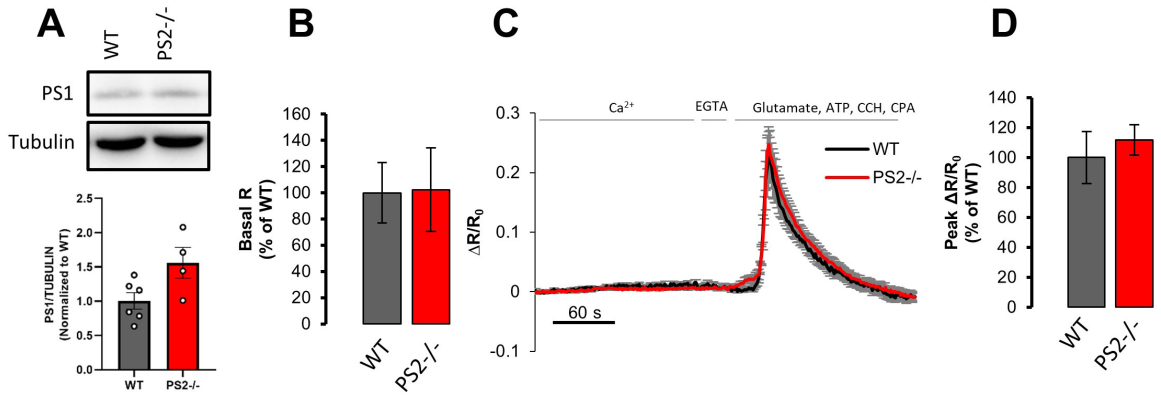

Figure S1. The lack of presenilin 2 (PS2) does not alter the cytosolic Ca^{2+} handling in hippocampal neurons. (**A**) Representative Western blot for PS1 expression level in wild-type (WT) and PS2-/- cortical neurons. The corresponding scatter plot shows the mean values±SEM; 6 independent cultures from WT and 4 independent cultures from PS2-/- cortical neurons. Each sample was run in duplicate. Values were first normalized to tubulin and then to those of WT samples. (**B**) The bar graph shows the mean basal R±SEM, as a percentage of WT, obtained in WT (gray, *n*=23 cells of 6 independent cultures) or PS2-/- (red, *n*=23 cells of 6 independent cultures) hippocampal neurons expressing the cytosolic Ca^{2+} probe D3mCerulean3+16. (**C**) Average traces of cytosolic Ca^{2+} kinetics in WT (black) or PS2-/- (red) hippocampal neurons expressing the cytosolic Ca^{2+} probe D3mCerulean3+16, bathed in mKRB and exposed to the mix of drugs described in figure 1B, after 20 seconds of EGTA (0.6 mM)-containing mKRB perfusion. Data are shown as mean±SEM of $\Delta R/R_0$ obtained in WT (gray, *n*=23 cells of 6 independent cultures) or PS2-/- (red, *n*=23 cells of 6 independent cultures) hippocampal neurons. (**D**) The bar graph shows the mean±SEM of cytosolic Ca^{2+} peak amplitude, as a percentage of WT, obtained in WT (gray) or PS2-/- (red) hippocampal neurons.

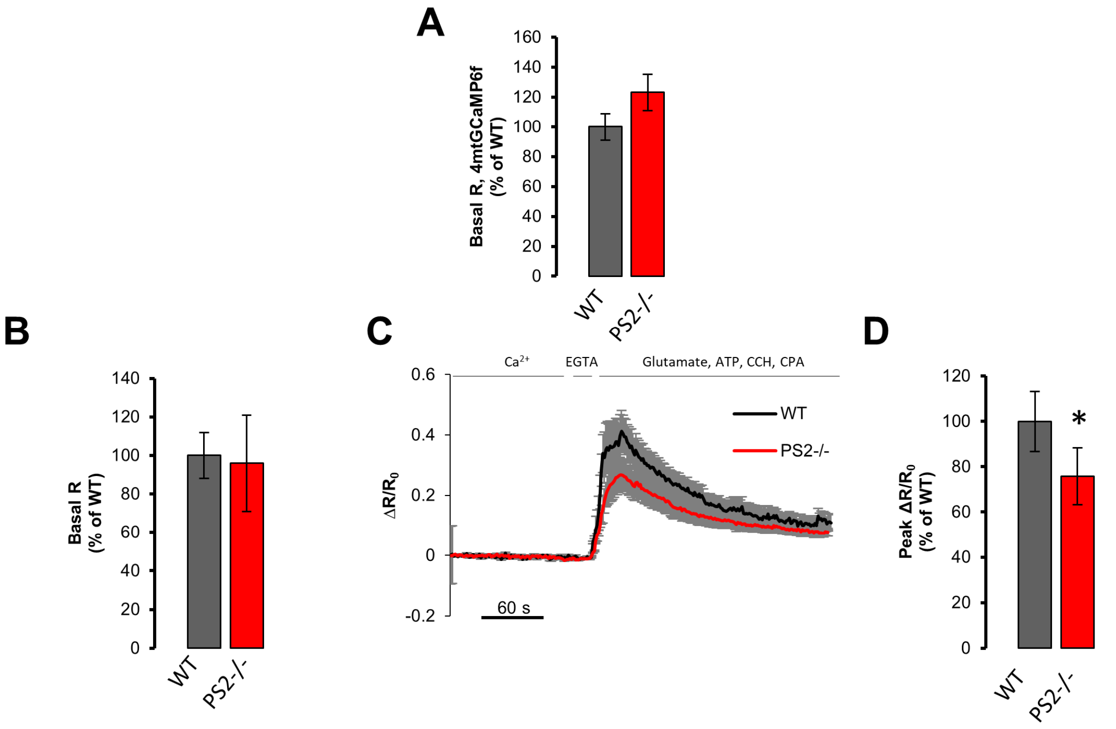

Figure S2. The lack of PS2 dampens mitochondrial Ca^{2+} uptake in hippocampal neurons. (**A**) The bar graph shows the mean basal R(473/410)±SEM of in WT (gray) or PS2-/- (red) cortical neurons expressing the mitochondrial Ca^{2+} probe 4mtGCaMP6f, as a percentage of WT, obtained in WT (gray, *n*=142 cells of 4 independent cultures) or PS2-/- (red, *n*=107 cells of 4 independent cultures) cortical neurons. (**B**) The bar graph shows the mean basal R±SEM, as a percentage of WT, obtained in WT (gray, *n*=12 cells of 7 independent cultures) or PS2-/- (red, *n*=25 cells of 5 independent cultures) hippocampal neurons expressing the mitochondrial Ca^{2+} probe 4mtD3mCerulean3+16. (**C**) Average traces of mitochondrial Ca^{2+} kinetics in WT (black) or PS2-/- (red) hippocampal neurons expressing the mitochondrial Ca^{2+} probe 4mtD3mCerulean3+16, bathed in mKRB and exposed to the mix of drugs described in Figure 1B, after 20 seconds of EGTA (0.6 mM)-containing mKRB perfusion. Data are shown as mean±SEM of $\Delta R/R_0$ obtained in WT (*n*=12 cells of 7 independent cultures) or PS2-/- (*n*=25 cells of 5 independent cultures) hippocampal neurons. (**D**) The bar graph shows the mean±SEM of mitochondrial Ca^{2+} peak amplitude, as a percentage of WT, obtained in WT (gray) or PS2-/- (red) hippocampal neurons.

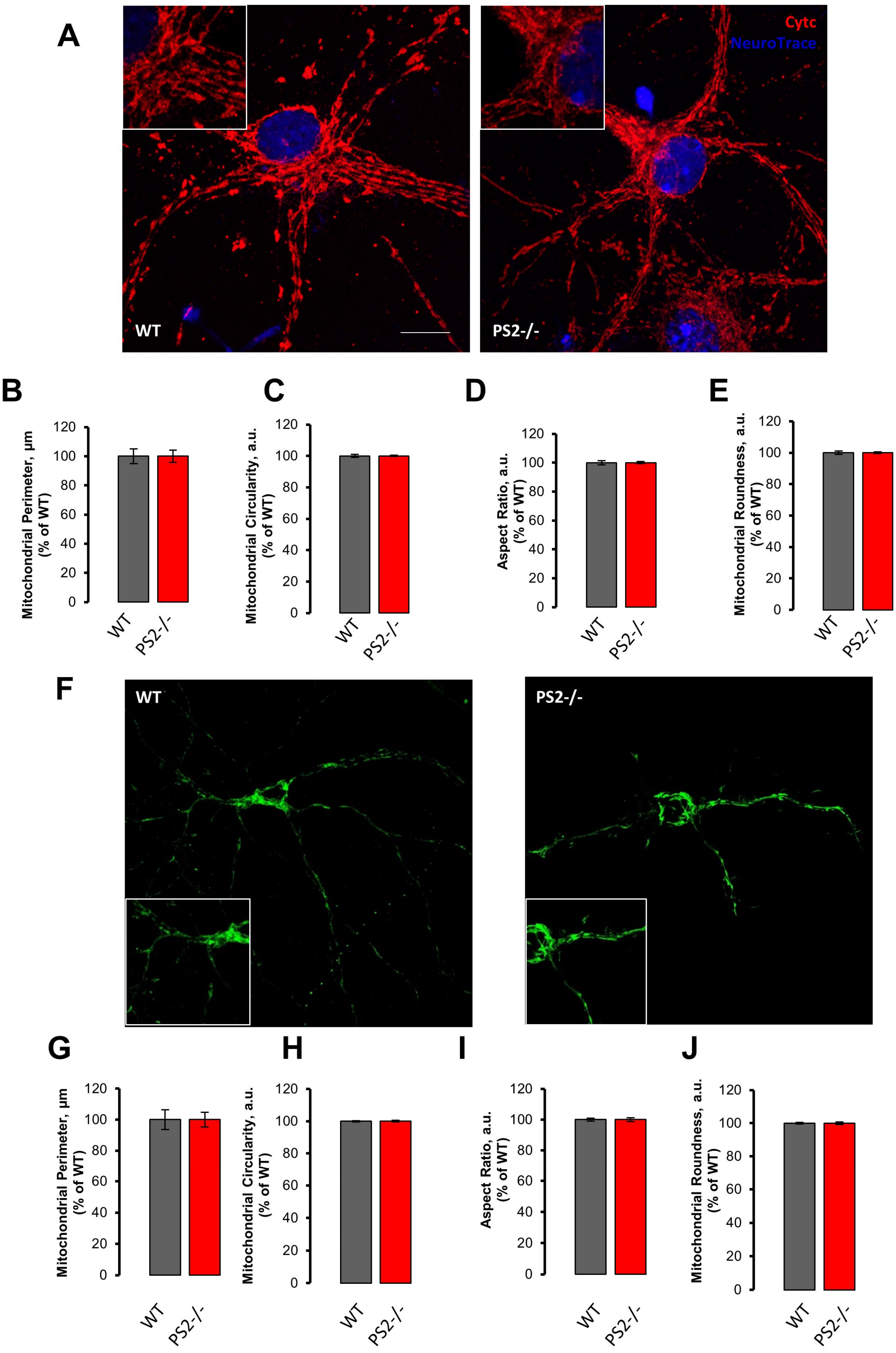

Figure S3. The lack of PS2 does not affect mitochondrial morphology. (**A**) Representative image of a cortical neuron from WT and PS2-/- mice, as indicated, with mitochondria labelled in red using an anti-cytochrome *c* antibody marked with Alexa 555, and the nucleus labelled in blue using NeuroTrace™. Highlighted boxes show cellular zones at higher magnification to better visualize the mitochondria shapes. Scale bar, 10 μm. The bar graphs show: (**B**) mitochondrial perimeter±SEM, (**C**) mitochondrial circularity values±SEM, and (**D**) mitochondrial aspect ratio±SEM, (**E**) mitochondrial

roundness values±SEM, as a percentage of WT, obtained in WT (gray) or PS2-/- (red) cortical neurons by immunofluorescence. The mean values were obtained from *n*=33 WT and *n*=27 PS2-/- neurons from 3 independent cultures. (**F**) Representative image of a cortical neuron from WT and PS2-/-mice infected with the mitochondrial probe 4mtD3mCerulean3+16. Highlighted boxes show cellular zones at higher magnification to better visualize the mitochondria shapes. Scale bar, 10 μm. The bar graphs show: (**G**) mitochondrial perimeter±SEM, (**H**) mitochondrial circularity values±SEM, (**I**) mitochondrial aspect ratio±SEM, and (**J**) mitochondrial roundness values±SEM, as a percentage of WT, obtained in WT (gray) or PS2-/- (red) cortical neurons by 4mtD3mCerulean3+16 expression. The mean values were obtained from *n*=44 cells of 3 independent cultures in both genotypes.

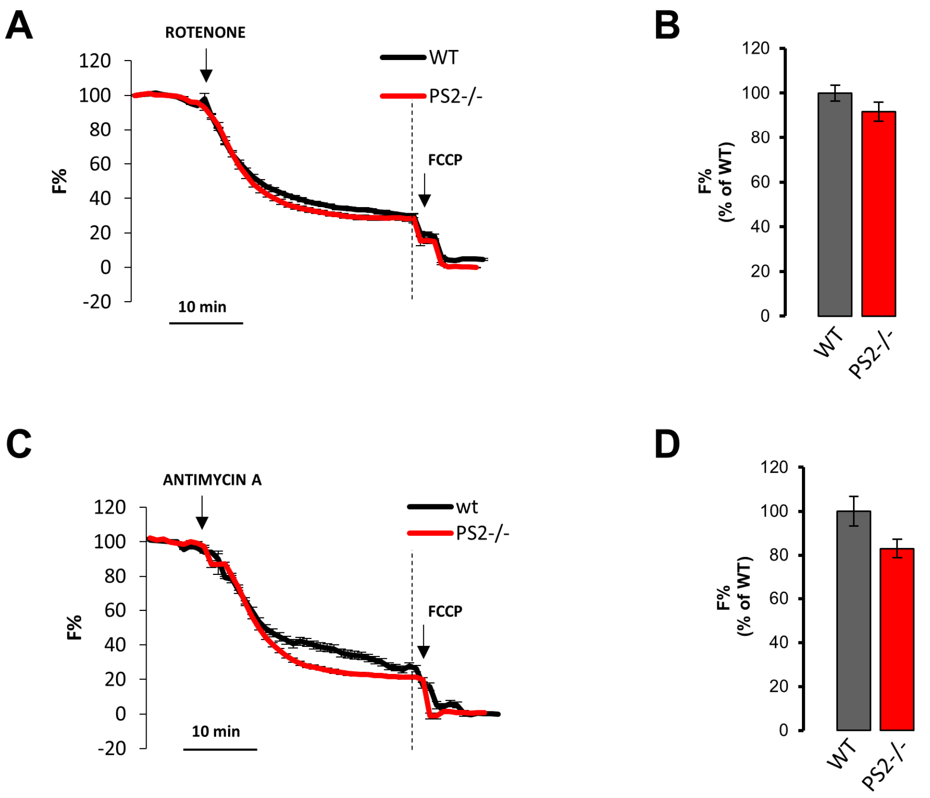

Figure S4. The lack of PS2 does not affect ΔΨm. (**A–C**) Average traces of WT (black) or PS2-/- (red) cortical neurons loaded with tetramethyl rhodamine methyl ester (TMRM) and bathed in K^+-based mKRB in the absence of external Ca^{2+} and exposed to rotenone (1 μM, **A**) or antimycin A (1 μM, **C**) for 30 minutes and carbonyl cyanide-4-(trifluoromethoxy)phenylhydrazone (FCCP) (10 μM) for 10 minutes. Data are shown as mean±SEM of F% obtained in WT (rotenone: *n*=165 cells of 8 independent cultures; antimycin A: *n*=123 cells of 8 independent cultures) or PS2-/- (rotenone: *n*=109 cells of 7 independent cultures; antimycin A: *n*=237 cells of 10 independent cultures) cortical neurons for antimycin experiments. (**B–D**) The bar graph shows the mean±SEM of F%, as a percentage of WT, obtained in WT (gray) or PS2-/- (red) cortical neurons upon rotenone (**B**) or antimycin A (**D**) addition, calculated at the time point indicated by the dashed line of panels **A** or **C**, respectively.

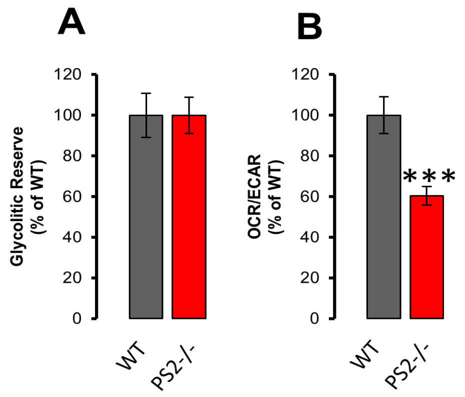

Figure S5. The lack of PS2 does not affect the glycolytic reserve, while it decreases Oxygen Consumption Rate (OCR)/Extracellular Acidification Rate (ECAR) ratio. The bar graph shows the mean±SEM, as a percentage of WT, obtained in WT (gray) or PS2-/- (red) cortical neurons of: (**A**) the glycolytic reserve (calculated as difference between ECAR after oligomycin addition and basal ECAR), and (**B**) the ratio between OCR and ECAR.

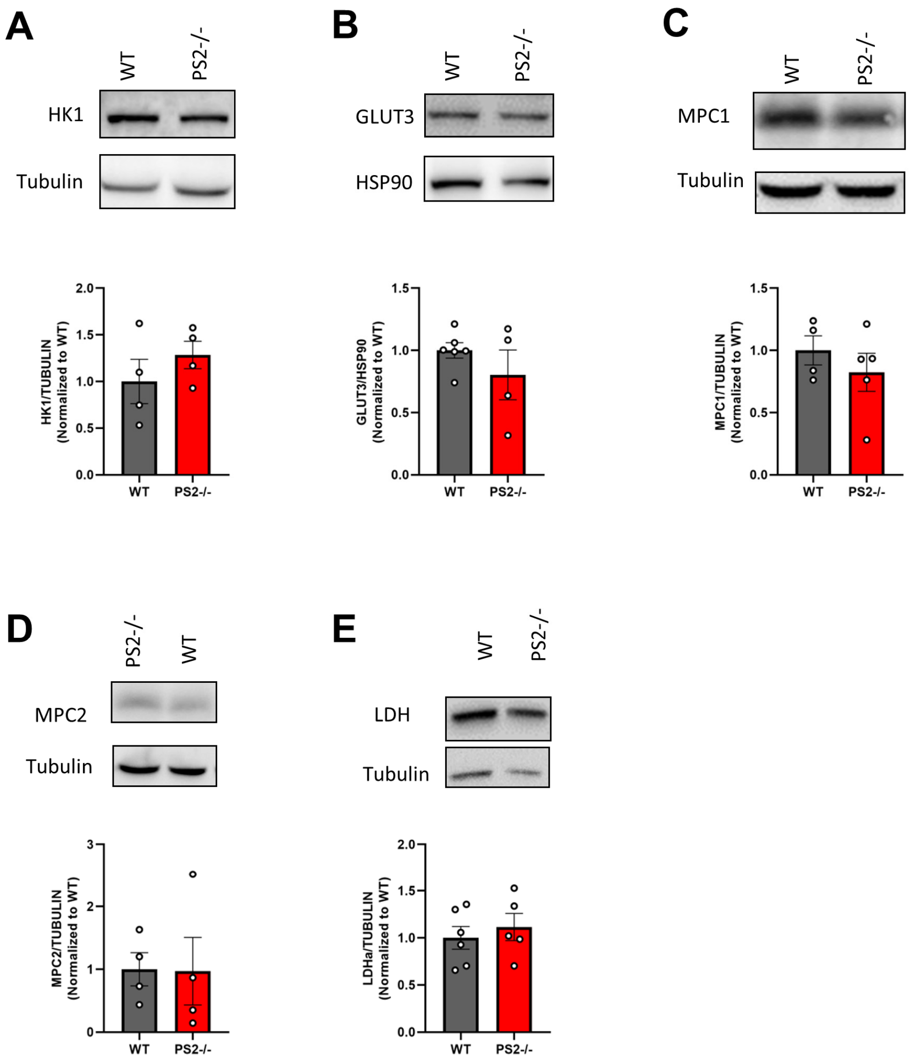

Figure S6. The lack of PS2 does not affect proteins involved in the regulation of glycolysis, Krebs cycle and oxidative phosphorylation. Representative Western blots of different proteins (as indicated in each panel) in WT and PS2-/- cortical neurons. Each sample was run in duplicate. The corresponding scatter plot shows the mean values±SEM. Values were first normalized to their internal housekeeping proteins (as indicated) and then to those of WT samples. Representative Western blot and quantification of: (**A**) HK1 protein levels of 4 independent cultures from WT mice and from PS2-/- mice; (**B**) GLUT3 protein levels of 6 independent cultures from WT mice and 4 independent cultures from PS2-/- mice; (**C**) MPC1 protein levels of 4 independent cultures from WT mice and 5 independent cultures from PS2-/- mice; (**D**) MPC2 protein levels of 4 independent cultures from WT mice and from PS2-/- mice; (**E**) LDH protein levels of 6 independent cultures from WT mice and 5 independent cultures from PS2-/- mice.
